# Identification of sepsis subtypes in critically ill adults using gene expression profiling

**DOI:** 10.1186/cc11667

**Published:** 2012-10-04

**Authors:** David M Maslove, Benjamin M Tang, Anthony S McLean

**Affiliations:** 1 Center for Clinical Informatics, Stanford University School of Medicine, Stanford, CA, USA; 2 Biomedical Informatics Training Program, Stanford University School of Medicine, Stanford, CA, USA; 3 Department of Intensive Care Medicine, Nepean Hospital and Nepean Clinical School, University of Sydney, Penrith, NSW 2750, Australia; 4 School of Public Health, Faculty of Medicine, University of Sydney, NSW 2006, Australia

**Keywords:** Sepsis, severe sepsis, septic shock, gene expression profiling, microarray analysis, biomedical informatics, critical care, intensive care

## Abstract

**Introduction:**

Sepsis is a syndromic illness that has traditionally been defined by a set of broad, highly sensitive clinical parameters. As a result, numerous distinct pathophysiologic states may meet diagnostic criteria for sepsis, leading to syndrome heterogeneity. The existence of biologically distinct sepsis subtypes may in part explain the lack of actionable evidence from clinical trials of sepsis therapies. We used microarray-based gene expression data from adult patients with sepsis in order to identify molecularly distinct sepsis subtypes.

**Methods:**

We used partitioning around medoids (PAM) and hierarchical clustering of gene expression profiles from neutrophils taken from a cohort of septic patients in order to identify distinct subtypes. Using the medoids learned from this cohort, we then clustered a second independent cohort of septic patients, and used the resulting class labels to evaluate differences in clinical parameters, as well as the expression of relevant pharmacogenes.

**Results:**

We identified two sepsis subtypes based on gene expression patterns. Subtype 1 was characterized by increased expression of genes involved in inflammatory and Toll receptor mediated signaling pathways, as well as a higher prevalence of severe sepsis. There were differences between subtypes in the expression of pharmacogenes related to hydrocortisone, vasopressin, norepinephrine, and drotrecogin alpha.

**Conclusions:**

Sepsis subtypes can be identified based on different gene expression patterns. These patterns may generate hypotheses about the underlying pathophysiology of sepsis and suggest new ways of classifying septic patients both in clinical practice, and in the design of clinical trials.

## Introduction

The protean illnesses of the ICU are syndromic in nature, defined by a number of clinical, laboratory and radiologic criteria, rather than specific pathologic findings. Examples of this include acute respiratory distress syndrome (ARDS), acute kidney injury (AKI) and sepsis. In such cases, the lack of specificity of the diagnostic criteria may lead to the inadvertent grouping together of physiologically disparate disease states under the same rubric [1,2]. By failing to account for the existence of subtypes, such syndromic definitions may have an averaging effect, which could account for negative or conflicting results from clinical trials [3]. Identifying syndrome subtypes is, therefore, an important objective, with the potential to significantly refine enrollment in randomized controlled trials, and tailor therapies in practice [2].

Gene expression microarray data may be useful in identifying sepsis subtypes based on differential expression of key genes [4]. In pediatric patients, unsupervised clustering methods have been used to identify sepsis subtypes based on gene expression profiles from whole blood, and have been shown to correlate with outcomes [5-7]. No such analysis, however, has been applied to adult cases. In this study, we present an analysis of gene expression profiles from adult patients with sepsis, in which subtypes are identified using bioinformatics techniques.

## Materials and methods

### Microarray data

Microarray data were obtained from two previously published, prospectively designed studies of gene expression in sepsis. Patient enrollment, data collection, RNA extraction and gene-expression profiling were carried out in the same manner for both studies, and are described in detail elsewhere [8,9]. Briefly, patients were recruited from the intensive care unit (ICU) of Nepean Hospital, Sydney, Australia. Neutrophils were isolated from blood samples taken within 24 hours of admission, and RNA extracted using guanidinium thiocyanate was converted to cDNA. Complimentary DNA derived from the RNA was fluorescently labeled, and hybridized to human oligonucleotide arrays consisting of 18,664 genes. Expression levels were determined by intensity of fluorescence captured by a laser scanner. The experimental design, RNA extraction and microarray experiments were all MIAME (minimum information about a microarray experiment)-compliant, and complete raw and normalized microarray data are available through the Gene Expression Omnibus (GEO) of the National Centre for Biotechnology Information (accession numbers GSE6535, and GSE5772) [10].

Data from two separate studies conducted using the same tissue and the same microarray platform were used. In both studies, sepsis was defined as the presence of systemic inflammatory response syndrome (SIRS) and infection, where the diagnosis of infection required the presence of clinical, as well as laboratory or pathological, evidence of infection. The first study included 72 critically ill patients, 55 of whom met diagnostic criteria for sepsis (derivation cohort). The second study included 94 critically ill patients, 71 of whom met diagnostic criteria for sepsis (validation cohort). The latter study included a larger number of missing values from gene expression profiling, and one patient from the sepsis group with > 80% missing data was removed.

### Identification of genetic subtypes

We used partitioning around medoids (PAM) clustering based on Euclidean distance, in order to identify sepsis subtypes within the gene expression profiles. From the derivation cohort, we identified the set of genes with the greatest differences in expression levels between subtypes and evaluated these as the gene signature.

In order to reduce the dimensionality of the dataset and improve the likelihood of discovering stable clusters, we used a multi-stage approach to feature selection. First, we searched Genbank for relevant genes using the terms "sepsis", "severe sepsis" and "septic shock". We then reduced the candidate genes to the intersection of the complete set of genes and the sepsis-specific set. Next, we carried out an enrichment step to identify the most discriminatory genes from within this subset, as well as the optimal number of clusters (*k*).

For the choice of cluster number, we randomly selected one-third of the candidate genes, and used these as the basis for PAM clustering over the range *k *= 2 to *k *= 10. We used the average silhouette width to evaluate the cohesiveness of the various clustering solutions [11]. The silhouette width is a combination of intra-cluster homogeneity and inter-cluster separation, for which higher values indicate better clustering. This procedure was repeated 100 times, and each time the value of *k *that generated the highest average silhouette width was recorded.

In order to increase the robustness of the cluster identification process and account for any inherent bias in the PAM clustering algorithm, this process was repeated using a hierarchical clustering algorithm based on a different similarity measure (Minkowski distance). We chose the value of *k *that most frequently produced the best result. The procedure to select the number of clusters was also repeated independently on the validation cohort, to determine whether the expression data in this group supported the same number of clusters as in the derivation group.

To identify a specific gene signature, we again used a process of randomly selecting one-third of the sepsis-specific genes, and used these as the basis for PAM clustering. One hundred cluster solutions based on random thirds were carried out. Each time the gene set producing the highest average silhouette width was added to a list of high-value genes. This process was repeated 100 times, after which we created a tally of the number of times each gene had been included in the high-value list. The 100 most frequently identified genes were taken as the enriched subset.

We used PAM clustering with the enriched subset of sepsis-specific genes to determine class labels for each of the patients in the derivation cohort. Using these labels, we then returned to the complete set of genes, and used significance analysis of microarrays (SAM) to identify genes that showed differential expression between groups [12]. This procedure assigns a score to each gene, based on the relationship for each observation between expression level, and that observation's class label. Genes with a q-value of 0, representing a very low likelihood of false discovery, were selected as the final gene set, and were used as the basis for an additional clustering step to determine the final class assignments.

### Cluster verification

As an additional verification step, we performed hierarchical clustering using Minkowski distance as a measure of similarity and Ward's method for agglomeration, and compared the cluster results to those obtained by PAM. We also carried out a number of statistical analyses to assess the internal validity of the clustering solutions derived from the above procedures. At each stage of enrichment, we evaluated the silhouette width of each cluster. We also carried out a bootstrapping cluster analysis to see how stable the solutions were, given the value of *k *identified in the preceding steps. We examined the distribution of individual gene expression values between cluster pairs, to determine whether these were bimodal, as would be expected if the clusters were distinct. This was done using the bimodality index [13], which yields a value representing the extent to which a distribution has two modes that are sufficiently separate. A bimodality index > 1.1 corresponds roughly to a distribution in which two modes are evident by visual inspection of a density plot. We also used principal components analysis (PCA) of the final clustering solution, and determined the bimodality index for the first principal component.

### Analysis of gene signature

For both the derivation and validation cohort, we used hierarchical clustering to identify the subset of genes that were differentially co-expressed between groups. We conducted a pathways analysis of this gene signature using the PANTHER classification system [14], with the entire human genome as background.

### Analysis of subtypes

We used the clustering solution derived from the method described above to identify sepsis subtypes within a second, independent dataset (validation cohort) that was based on the same microarray platform as the first. We limited the genes in this dataset to those that were included in the gene signature. To classify the patients in the validation cohort, we determined which of the derivation medoids they were closest to, and assigned them to the corresponding subtype.

Using these labels, we evaluated a limited number of clinical attributes using Fisher's exact test and Student's *t*-test. To investigate the potential role of genetic differences in accounting for some of the negative or conflicting clinical evidence for sepsis therapies, we looked at genes implicated in the action of a select group of drugs that have been studied in large-scale randomized controlled trials (RCTs) in sepsis. Using the Pharmacogenomics Knowledge Base (PharmGKB) [15], we identified genes that play a role in the action or metabolism of hydrocortisone, vasopressin, norepinephrine and drotrecogin alpha. We used GeneMania [16] to expand each of these gene sets by including up to 20 other genes that shared protein domains, physical interactions, pathways, or expression patterns with the original query set. We then used the results of SAM to determine if these genes showed differential expression between sepsis subtypes, reporting those with a high degree of statistical significance, indicated by a q-value of 0.

All analyses were performed using the R software environment for statistical computing and graphics, with functions from the samr, clValid, cluster, e1071 and ClassDiscovery libraries [17].

## Results

The Genbank search returned a total of 450 unique genes, 365 of which were included in the microarray platform. Using this subset of genes, the best silhouette values were achieved with a value of *k *= 2, regardless of whether PAM or hierarchical clustering was used. Results for the validation cohort were similar.

**Figure 1:**
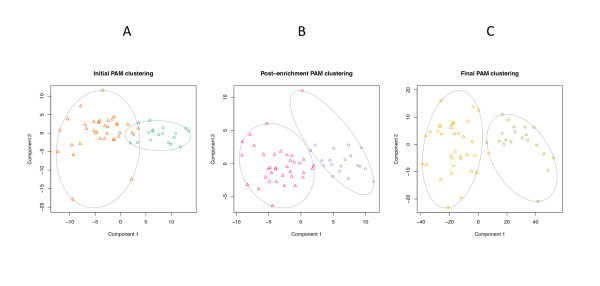
**Results of PAM clustering through successive enrichment stages**. In each plot, the patients are plotted within a two-dimensional space representing the greatest proportion of the variation in the dataset. The points in the first plot are colored according to the cluster assignments from the initial solution based on 365 sepsis-related genes found in Genbank. The colors in the second and third plots reflect the clustering from the preceding step. The symbols in each plot are determined by the results of the clustering at that stage. (**A**) Initial clustering based on the sepsis-specific genes found in Genbank. (**B**) Results of clustering following the 100-fold gene enrichment step. (**C**) Clustering based on the genes that were found to show differential expression after the SAM enrichment step.

Graphical representations of the derivation clusters through successive stages of enrichment are shown in Figure 1. Initially, there were 19 patients in cluster 1 and 36 patients in cluster 2, with an average silhouette width of 0.1. After the 100-fold enrichment step, the average silhouette width increased to 0.2, with 20 patients in cluster 1 and 35 patients in cluster 2. After SAM enrichment, there were 21 patients in cluster 1 and 34 patients in cluster 2, with an average silhouette width of 0.3.

**Figure 2:**
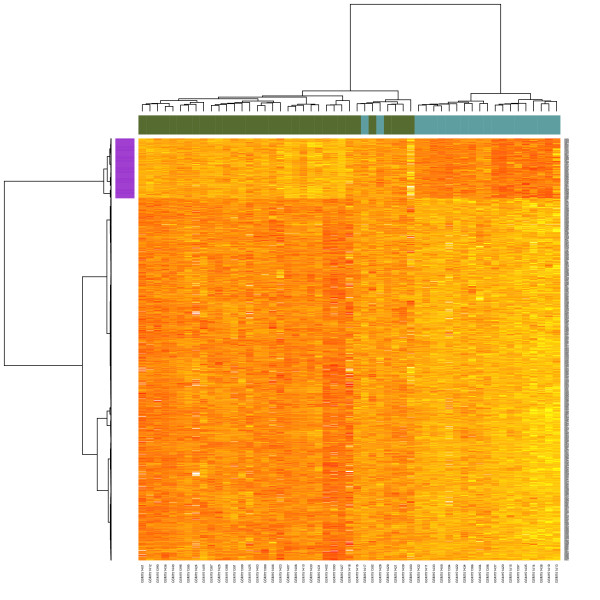
**Heatmap showing the results of hierarchical clustering of the derivation dataset**. Clustering is based on the genes identified by the enrichment process described. Results are based on Minkowski distance and Ward's method of agglomeration. The color bars at the top of the heatmap represent the cluster assignments determined by PAM clustering. The colored bar next to the row dendrogram shows the co-expressed genes that were used as the final gene signature.

**Figure 3:**
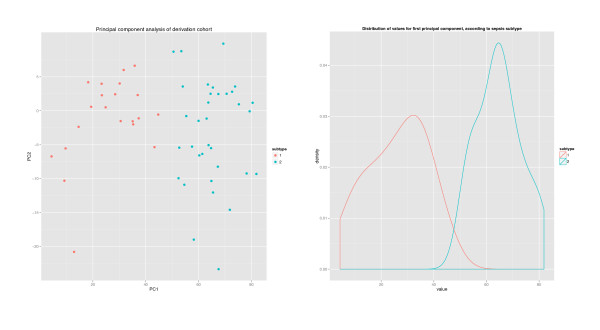
**Principal components analysis of the gene expression data**. Patient gene expression values plotted within the first two principal components (left). Distribution of values for the first principal component, according to subtype (right).

The results of hierarchical clustering (Figure 2) reveal a difference in class assignment between the two clustering methods for two patients. Bootstrapping cluster analysis showed that clustering with *k *= 2 was stable (Additional file 1). As expected, this was seen for the final cluster solution that was based in part on genes known to have differential expression between groups. However, this was also seen for the clusters derived from the genes identified by the Genbank search, prior to their subsetting based on expression differences. Bimodal indices were greater than 1.1 for approximately 28% of the 1,256 genes identified in the SAM enrichment step (Additional file 2). PCA showed that the two subtypes were separable in the first principal component, which accounted for 45% of the variance (Figure 3). The distribution of values yielded a bimodal index of 1.85, suggesting the presence of two distinct modes.

**Figure 4:**
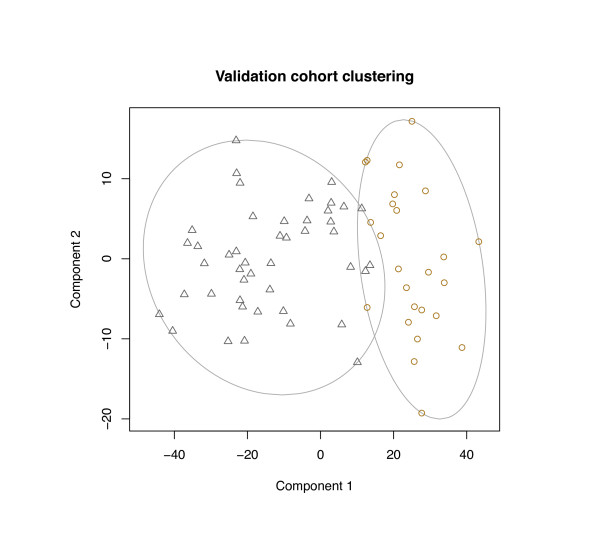
**Validation cohort clustering**. Clusters resulting from assignment of the validation cohort samples to the closest derivation medoid.

Using the clustering medoids derived in the first step, the observations in the validation cohort were assigned class labels based on the closest medoid (Euclidean distance). Internal measures showed cluster stability similar to that achieved with the derivation cohort, including an average silhouette width of 0.26 (Figure 4).

Hierarchical clustering revealed 178 co-expressed genes in the derivation cohort, and 171 co-expressed genes in the validation cohort. All but one of the validation genes was also found in the derivation set, and the 170-gene intersection was taken as the gene signature (Additional file 3). Pathway analysis (Table 1) revealed this signature to be enriched for two cellular processes relevant to sepsis and shock, namely inflammation mediated by chemokine and cytokine signaling pathways, and Toll receptor signaling pathway. The subtype 1 pattern showed increased expression of these genes relative to subtype 2.

**Table 1:** Pathway analysis using the gene signature discovered during the identification of sepsis subtypes

Pathway	*P*-value	Bonferonni
Inflammation mediated by chemokine and cytokine signaling pathway	2.70E-08	0.0000048
Toll receptor signaling pathway	7.66E-05	0.0135
T cell activation	4.75E-03	0.835
p38 MAPK pathway	5.38E-03	0.946
JAK/STAT signaling pathway	7.59E-03	1
Beta3 adrenergic receptor signaling pathway	0.02	1
Integrin signalling pathway	0.02	1
B cell activation	0.02	1
Interferon-gamma signaling pathway	0.02	1
Opioid prodynorphin pathway	0.02	1
Opioid proenkephalin pathway	0.02	1
5HT4 type receptor mediated signaling pathway	0.02	1
Opioid proopiomelanocortin pathway	0.03	1
Salvage pyrimidine deoxyribonucleotides	0.03	1
Heterotrimeric G-protein signaling pathway-Gi alpha and Gs alpha mediated pathway	0.04	1
Nicotinic acetylcholine receptor signaling pathway	0.04	1
Parkinson disease	0.04	1
Beta2 adrenergic receptor signaling pathway	0.04	1
Beta1 adrenergic receptor signaling pathway	0.04	1
5HT1 type receptor mediated signaling pathway	0.04	1

**Figure 5:**
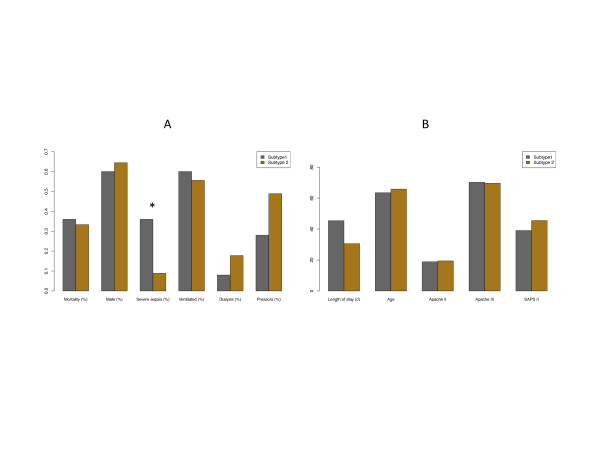
**Clinical features of the validation cohort**. Differences in clinical features between the two subtypes. Asterix signifies *P *< 0.05. LOS, length of stay.

The clinical differences between subtypes within the validation cohort dataset are shown in Table 2 and Figure 5. There were more patients diagnosed with severe sepsis in subtype 1 (36%) than in subtype 2 (9%). The proportions of patients with septic shock were similar. Analysis of expression levels of relevant pharmacogenes revealed a number of statistically significant differences between subtypes, ranging from 1.3- to 3-fold differences in expression (Table 3). These included genes were implicated in pathways important to drotrecogin alpha, vasopressin, hydrocortisone and norepinephrine.

**Table 2:** Comparison of clinical attributes between the two sepsis subtypes defined by gene expression profiles

Clinical attribute	Subtype 1	Subtype 2	*P*-value
Mortality (%)	36	33	1
Male (%)	60	64	0.80
Severe sepsis (%)	36	9	0.009
Septic shock (%)	44	64	0.13
Ventilated (%)	60	56	0.80
Dialysis (%)	8	18	0.31
Vasopressors (%)	28	49	0.13
Gram positive (%)*	62	42	0.26
Gram negative (%)*	57	71	0.38
Length of stay (days)	45	31	0.23
Age	63	66	0.56
APACHE II	19	19	0.78
SAPS II	39	45	0.10
APACHE III	70	70	0.93

*Percentage of patients who were culture positive. Four patients from each subtype had both Gram positive and Gram negative organisms isolated.

**Table 3:** Differences in expression of relevant pharmacogenes between the two sepsis subtypes

Gene ID	Fold change
drotrecogin alpha	
TFPI	1.74
SERPINB2	1.61
CP	1.52
GGCX	1.49
SERPIND1	1.58
SERPINB6	1.82
SERPINE1	1.43
THBD	0.53
F5	0.48
Vasopressin	
GNG11	1.73
GNG5	1.43
GNAQ	0.58
Hydrocortisone	
ALOX5	0.34
ANXA1	0.64
Norepinephrine	
NNMT	1.32
MOXD1	1.42

Fold change based on significance analysis of microarrays (SAM), with q-value for each gene listed equal to 0. Values shown represent expression ratio of type 2 relative to type 1.

## Discussion

Sepsis continues to be a major public health concern. With only supportive measures showing benefit in clinical trials and no specific syndromic therapies available, mortality from this condition remains high. Although sepsis is a multifaceted pathophysiologic state involving multiple organ and cellular systems, it is most often diagnosed, treated and studied based on a clinical definition incommensurate with its complexity.

Subtypes of disease can be defined in many ways, including by epidemiologic, clinical, pathological, genetic and molecular characteristics. In this study, we used microarray data derived from the neutrophils of patients diagnosed with sepsis in order to determine if more than one distinct gene expression pattern exists among them. Related approaches have been used previously, most notably in cancer biology, to identify subtypes of diffuse large B-cell lymphoma [18], breast cancer [19], lung cancer [20] and melanoma [21]. We chose to examine the genetic underpinnings of sepsis because it is a highly complex, multi-organ process that may defy reduction to traditional clinical parameters. Moreover, the differences in clinical course and response to therapy in sepsis are not fully explained by clinical characteristics alone.

We identified two sepsis subtypes based on gene expression profiles among patients meeting traditional diagnostic criteria for sepsis. We used multiple objective measures with two different clustering algorithms, to validate the number of clusters in the cohort, as well as a multifaceted approach to identify the subset of genes that were most discriminatory for classification purposes. This method combines domain knowledge derived from Genbank with an iterative approach designed to produce a subset of genes that was enriched for use in cluster identification. Importantly, we then returned to the complete set of genes and identified a larger subset based on differential expression, thus maximizing the opportunity to derive new knowledge about the role in sepsis of genes not previously associated with this disease. Our results show increasing cluster stability with each successive step of the gene signature discovery process. Moreover, we observed excellent agreement between clustering methods.

The subtype 1 gene expression profile was characterized by significantly increased expression of genes involved in inflammatory and Toll receptor mediated signaling pathways, and was associated with a higher prevalence of severe sepsis. These signaling pathways have been shown previously to be dynamically expressed in the course of sepsis, and to correlate with sepsis severity [22-24]. Other clinical attributes, including age, severity of illness scores, mortality and need for organ support, were similar between the two subtypes.

Expression differed significantly for a number of pharmacogenes of drugs found to have inconsistent effects in severe sepsis and septic shock. This heterogeneity of pharmacogene expression may have contributed to the negative results of large-scale RCTs, as only a subgroup of the participants may have responded to the treatment under investigation. In particular, the two-fold difference between subtypes in the expression of Factor V could have a significant impact on the efficacy of drotrecogin alpha, which targets this protein specifically.

We also observed a three-fold difference in the expression of ALOX5, a key component in the metabolism of leukotrienes that has been shown in mouse knockout models to worsen sepsis-induced multiple organ injury, and that is also mediated by glucocorticoid activity [25]. More recently, the combined COX-2 and ALOX5 inhibitor flavocoxid has shown significant efficacy in a mouse cecal ligation and puncture model of sepsis [26]. This compound, already used in the United States for the treatment of osteoarthritis, therefore shows promise as a potential therapy for sepsis. Our results suggest that patients may respond differently to this agent depending on their gene expression pattern. Stratifying patients according to gene expression subtype might, therefore, be one way of increasing the likelihood of obtaining meaningful results from future clinical trials of this agent.

Analysis of the gene signature also revealed significant differences between subtypes in the levels of gene expression for key pathways, including cytokine and Toll receptor mediated signaling pathways, that play central roles in the pathogenesis of sepsis [27,28]. This result may also be of therapeutic importance, as novel agents targeting Toll-like receptor pathways are being investigated for the treatment of sepsis [29]. Differential expression of genes in the target pathway among patients meeting clinical enrollment criteria could theoretically predispose such trials to heterogeneous treatment effects.

A study similar to ours by Wong *et al*. identified three molecularly distinct subtypes in pediatric sepsis, one of which was associated with higher severity of illness scores, and increased mortality [5]. Our study differs from this work in a few important ways. First, there are important differences in the pathophysiology of sepsis between children and adults that could lead to differences in gene expression profiles between these two groups [30]. Second, our method for determining the optimal number of subtypes was based on internal metrics of clustering success, rather than an *a priori *decision. Third, we used both PAM and hierarchical clustering, rather than K-means clustering, which may have different performance characteristics depending on the nature of the dataset in question. Fourth, we used gene expression data derived from neutrophils, while the study by Wong *et al*. used whole blood, which reflects expression from all leukocyte subtypes, weighted by their relative abundance at the time of sampling [31]. Lastly, our initial clustering was based on a subset of genes known from the literature to have relevance to sepsis, with subsequent clustering based on refinements of this gene signature by an iterative enrichment process. In the case of Wong *et al*., clustering was carried out on a subset of genes chosen based on differences in expression levels between patients with sepsis and non-sepsis controls. This latter approach has the potential to exclude genes that may be important in differentiating sepsis subtypes, rather than differentiating sepsis from controls.

Our results highlight the complexity and heterogeneity of sepsis at the molecular level, a finding in keeping with those of a recent systematic review on the subject [32]. Its strengths include the use of separate derivation and validation cohorts, the use of objective measures of internal cluster validity, and the use of two different clustering algorithms. Linkage to clinical data helped to characterize the validation cohort in terms of demographic characteristics and outcomes. We also used an approach with both knowledge-based and algorithmic components in order to reduce the feature space within which observations were clustered.

There are a number of limitations that must be addressed. First, the microarray data used in this analysis were obtained from neutrophils collected within 24 hours of admission to the ICU. While it has been suggested that the tissue used and timing of microarray analysis could have a significant impact on gene expression studies in sepsis [33], the experimental conditions were similar for all patients and for both cohorts, so that differences between individual patients should be minimized. Nonetheless, gene expression profiles are known to change rapidly in the early stages of injury and sepsis, and may in fact follow a trajectory between different states of immunostimulation [33,34]. Though the subtypes identified in our study showed good separation, they were not perfectly distinct. One possible interpretation of this result is that the overlap reflects sampling of patients in transitional states.

Second, the initial gene subset selection based on the Genbank search may have diminished the opportunity to discover expression differences among genes not otherwise known to be related to sepsis. We aimed to mitigate this effect by returning to the complete gene set prior to the final enrichment step, so as to allow the inclusion of any gene represented in the array.

Third, we did not collect data regarding ethnicity, which might affect gene expression levels and act as a confounding factor [35], or drug exposure, which would have been valuable in further exploring the differences in pharmacogene expression between subtypes.

Lastly, we note that clustering algorithms applied to microarray data must be used with caution, as these will invariably identify clusters [36]. Knowing whether such clusters reflect truly distinct subtypes, rather than artifacts of the datasets, remains a challenge to unsupervised machine learning methodologies in general. Nonetheless, an approach similar to ours has been used successfully in the past in other domains, including cancer [18-21,37] and Parkinson's disease [38], as well as in pediatric sepsis [5-7]. To guard against false cluster discovery, we employed an objective measure of cluster validity, and reproduced the result using two different clustering algorithms, in two independent datasets. Furthermore, we believe the existence of two separate clusters to be biologically plausible, insofar as the gene signature used to distinguish them includes a number of genes and pathways known to be important in the pathophysiology of sepsis and inflammation.

Our results are based on retrospective data and exploratory data analyses and, as such, cannot definitively prove the existence of non-overlapping genetic subtypes, nor define a gene signature for clinical use in the treatment of sepsis. Rather, our study is preliminary in nature and intended to be hypothesis generating.

Further gene expression studies of sepsis should focus not only on differentiating sepsis from controls, but sepsis subtypes as well. In this endeavor, a rich clinical database that includes information regarding patient ethnicity, drug exposure, and status at the time of sample collection, will assist in the interpretation of findings, and the conceptualization of subtypes in clinical practice. Furthermore, the results of pharmacogene expression analysis may be important in planning future RCTs of sepsis therapies, and in guiding treatment for patients with severe sepsis and septic shock.

## Conclusions

We present a novel method for the identification of molecularly distinct sepsis subtypes based on gene expression profiling in critically ill adults. We identified two subtypes that showed significant differences in the expression of genes related to well known sepsis pathways and therapeutics, and were associated with sepsis severity. Our results may help to explain negative or conflicting results in clinical trials of sepsis therapies, in which patients with heterogeneous genetic responses to the treatment in question may be inadvertently grouped together. As the availability of microarray-based diagnostics increases, their use in stratifying patients enrolled in sepsis trials should be explored.

## Key messages

• A lack of specificity of the diagnostic criteria for sepsis may lead to the inadvertent grouping together of physiologically disparate disease states into the same category.

• We used novel bioinformatics methods that combine domain knowledge and cluster analysis to identify two subtypes of sepsis based on gene expression profiles in critically ill adults.

• There were a number of significant differences between subtypes, including the prevalence of severe sepsis, as well as differences in the level of expression of genes relating to known sepsis pathways, and pharmacogenes relevant to sepsis therapies.

• These differences may explain in part the inconsistent results seen in sepsis clinical trials, and suggest new ways of stratifying patients in the future.

## Abbreviations

AKI: acute kidney injury; ARDS: acute respiratory distress syndrome; GEO: Gene Expression Omnibus; ICU: intensive care unit; MIAME: minimum information about a microarray experiment; PAM: partitioning around medoids; PCA: principal components analysis; RCTs: randomized controlled trials; SAM: significance analysis of microarrays; SIRS: systemic inflammatory response syndrome

## Competing interests

The authors declare that they have no competing interests.

## Authors' contributions

DM conceived of the study, performed statistical analyses, interpreted the data and drafted the manuscript. BT collected the data, carried out the initial microarray analyses, interpreted the data and revised the manuscript. AM collected the data, carried out the initial microarray analyses and interpreted the data. All authors read and approved the final manuscript.

## Supplementary Material

Additional file 1 **Bootstrapping cluster analysis**. Bootstrapping analysis of the derivation cohort with k=2, and 200-fold re-sampling. Hierarchical clustering was used, with Euclidean distance and Ward’s method for agglomeration. Color map values range from pure blue (the?samples are in the same branch 0% of the time) to pure yellow (the samples are in the same branch 100% of the time). (A) Result using the?initial gene set derived from Genbank. (B) Results following the gene enrichment stages. Analysis carried out in R using the ClassDiscovery package. Click here for file

Additional file 2 **Distributions of gene expression values**. Density plots for the 50 genes with highest bimodal index. Click here for file

Additional file 3 **Sepsis subtype gene signature **. Gene signature derived from the overlap of co-expressed genes in the derivation and validation cohorts. Click here for file
