# Supplementary material for: Identification of sepsis subtypes in critically ill adults using gene expression profiling
Source: Crit Care. 2012 Oct 4;16(5):R183. doi: 10.1186/cc11667 (PMC3682285; doi:10.1186/cc11667)
Supplement: Additional file 1 — Bootstrapping cluster analysis. Bootstrapping analysis of the derivation cohort with k=2, and 200-fold re-sampling. Hierarchical clustering was used, with Euclidean distance and Ward’s method for agglomeration. Color map values range from pure blue (the?samples are in the same branch 0% of the time) to pure yellow (the samples are in the same branch 100% of the time). (A) Result using the?initial gene set derived from Genbank. (B) Results following the gene enrichment stages. Analysis carried out in R using the ClassDiscovery package. [file cc11667-S1.PDF]

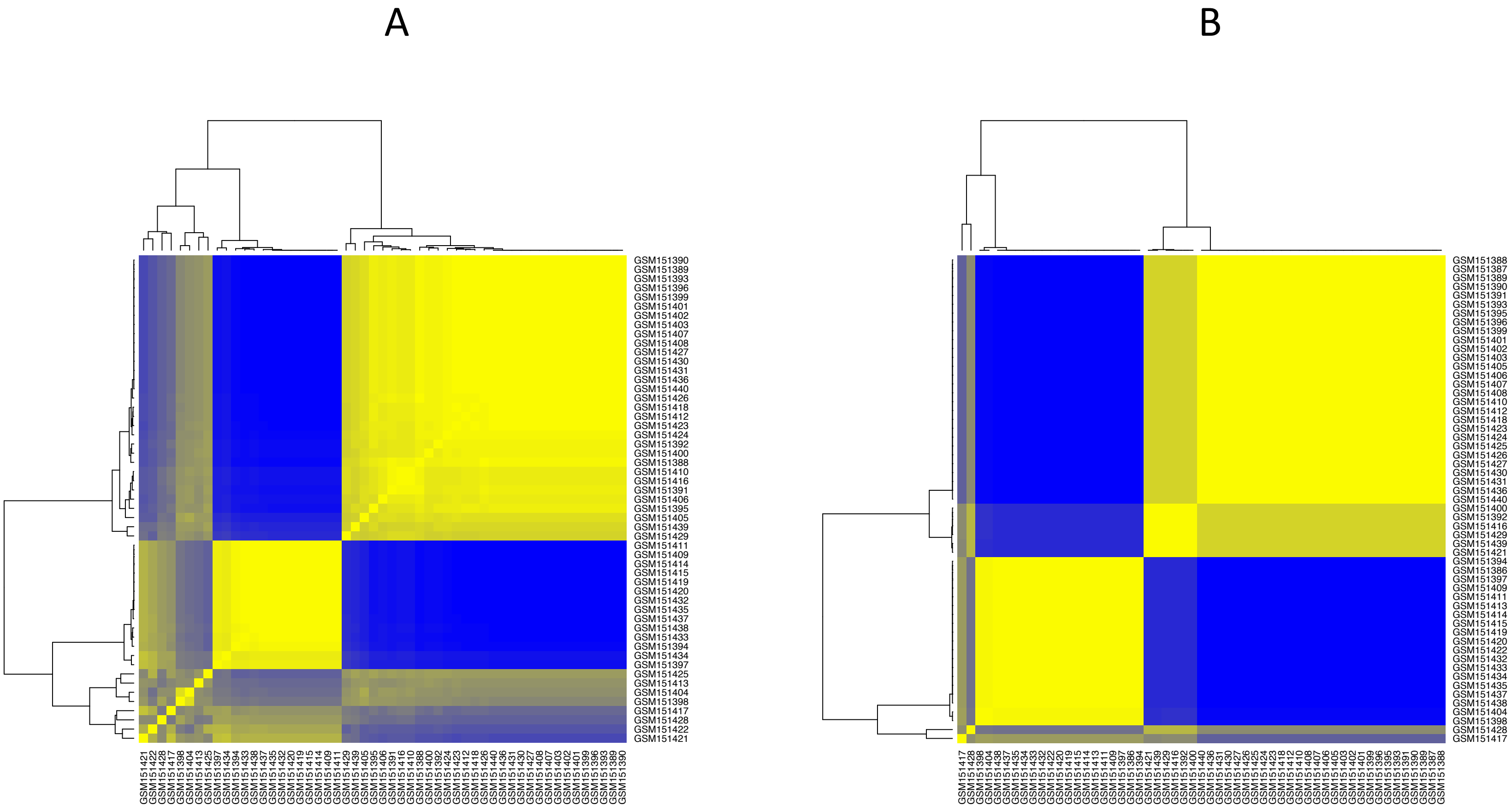

Supplementary Figure 1. Bootstrapping cluster analysis of the derivation cohort with *k* = 2, and 200-fold re-sampling. Hierarchical clustering was used, with euclidean distance and Ward's method for agglomeration. Color map values range from pure blue (the samples are in the same branch 0% of the time) to pure yellow (the samples are in the same branch 100% of the time). (A) Result using the initial gene set derived from Genbank. (B) Results following the gene enrichment stages. Analysis carried out in R using the ClassDiscovery package.
