# Supplementary figures and images for: Identification of sepsis subtypes in critically ill adults using gene expression profiling
Source: Crit Care. 2012 Oct 4;16(5):R183. doi: 10.1186/cc11667 (PMC3682285; doi:10.1186/cc11667)

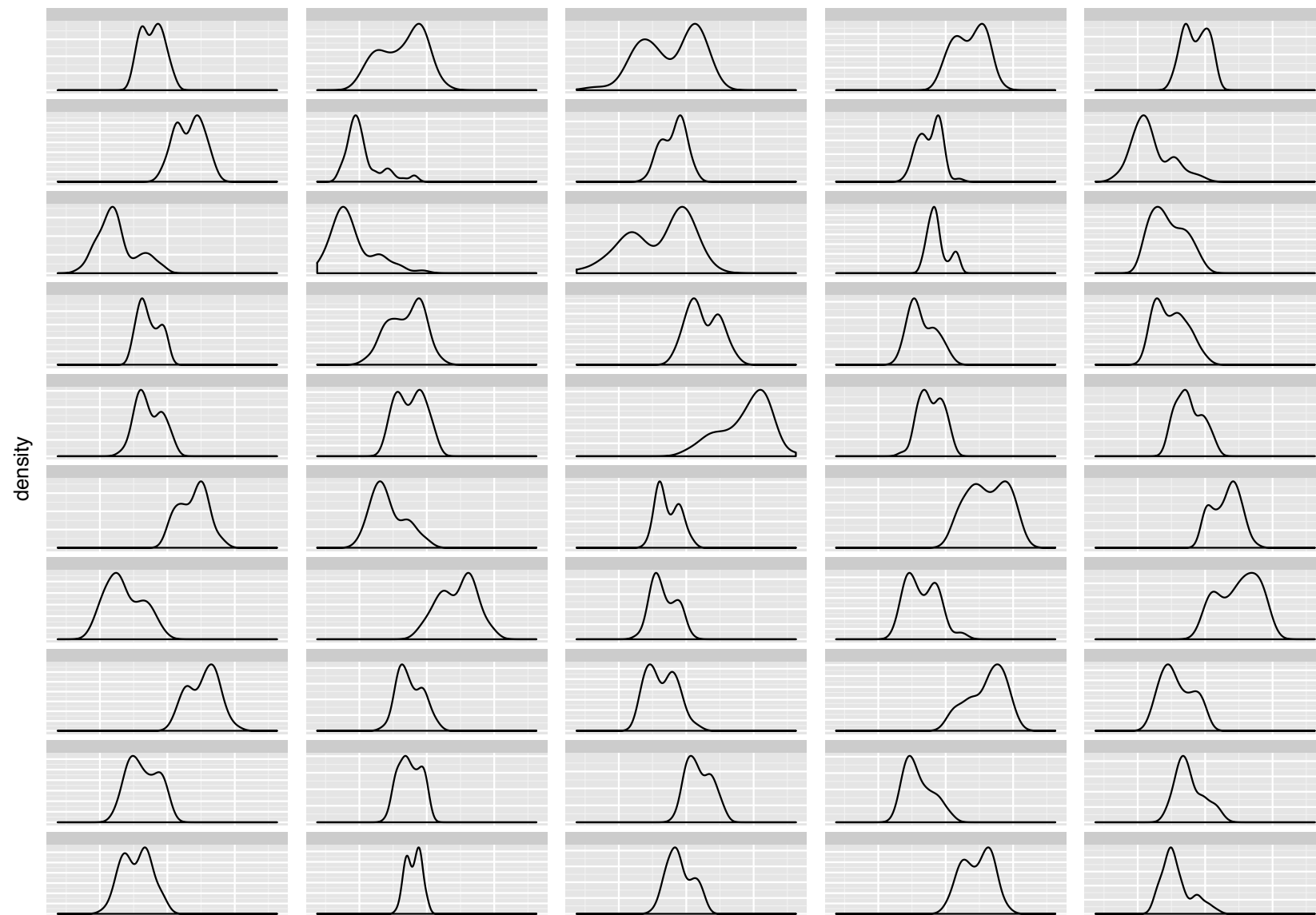

**Supplementary Figure 2.** Density plots for the 50 genes with highest bimodal index.

Supplement: Additional file 2 — Distributions of gene expression values. Density plots for the 50 genes with highest bimodal index. [file cc11667-S2.PDF]
