# Supplementary material for: Identification of sepsis subtypes in critically ill adults using gene expression profiling
Source: Crit Care. 2012 Oct 4;16(5):R183. doi: 10.1186/cc11667 (PMC3682285; doi:10.1186/cc11667)
Supplement: Additional file 3 — Sepsis subtype gene signature . Gene signature derived from the overlap of co-expressed genes in the derivation and validation cohorts. [file cc11667-S3.PDF]

XPO6
MTMR3
NBEAL2
MEGF9
ALPL
PADI2
ABHD5
AF007147
AQP9
RAB3D
CD300A
AF035318
TLR5
RGS18
MS4A6A
ARHGAP9
SIGLEC9
NOTCH1
AJ251235
PRAM1
FGD3
C9ORF72
MRVI1
SEMA4A
ZNF408
TREML2
AKNA
C10ORF54
AK024629
PTEN
NLRC5
SPATA13
TMEM149
PLBD1
OBFC2A
LRRK2
AL080095
LRRC4B
AL137709
EIF4E3
NLRC4
CLEC7A
FCGR1A
ARHGDIB
CDA
TFEB

CD53
IL1RN
SERPINB1
ARG1
ITGB2
NCF1
WAS
IFNGR1
NCF2
CD14
ITGAM
CXCR1
SELL
ALOX5
PTAFR
TNFRSF1A
ADAM8
ADCY7
MAPK14
FPR2
ALOX5AP
RHOG
C5AR1
CD37
CDKN2D
SLC31A2
ACSL1
FPR1
H3F3A
HAL
HK3
HSPA6
KCNJ15
LAIR1
MXD1
MMP8
MNDA
PTPRC
PYGL
RAC2
RGS2
SPI1
DYSF
NDST2
NCOA1
STX11

S1PR4
SIGLEC5
IL18R1
B2M
C3AR1
PTK2B
STX3
STK17B
CCNG2
PHC2
GALNT3
IL16
PFKFB3
IL1R2
VNN2
VNN1
MGAM
LPAR2
CCPG1
C1ORF38
LITAF
EI24
CREB5
ANXA3
CEBPB
FGR
HCLS1
INPP5D
S100A12
SDCBP
NAMPT
PLXNC1
DHRS9
IGSF6
GAS7
MTF1
TLR6
MRVI1
TRIM22
CSF2RA
NFE2
EVI2B
PTPRE
CELF2
TNFSF13B
LAPTM5

RIPK3
TNFAIP6
IRAK3
KLHL2
GCA
PADI4
NCF4
RAC2
LAT2
IL1RAPL1
GPR160
NM_014549
SLC40A1
FAM65B
NM_014863
CARD8
LY96
PTPN22
CRLF3
SH3GLB1
BIN2
SEPX1
TDP2
NOTCH1
NM_018623
RBM47
CD177
MBOAT7
SLC25A37
U32248
MYO1F
CR1
